# Golden

**Published:** 1886-11

**Authors:** J. W. Clowes

**Affiliations:** New York City


### GOLDEN.

BY J. W. CLOWES, D. D. S., NEW YORK CITY.

READ BEFORE THE CENTRAL DENTAL ASSOCIATION OF NORTHERN NEW JERSEY.

Gold has the significance of value. The "Golden bowl" of life has a vital issue—The "Golden rule" is our perfect law—"Golden harvests" are garners of plenty, and "golden opportunities" the mediums of success! Gold is an excellent material for filling teeth, but *its efficiency depends chiefly upon the manner of its use.* The first gold fillings of which I have any recollection, were seen in some attempts upon teeth of negroes, who came before an operative clinic of the Baltimore College of Dental Surgery, in 1840. Each student was allowed to take his turn in excavating and filling a cavity with gold. I was the third novitiate on trial, and, having looked upon my predecessors as blunderers (because their fillings would not stick without assistance), begged not to be helped over any difficulty that might occur. The confidence of youth encompassed me, and made the filling of a cavity seem so simple and easy that I felt sure of success. But my failure proved worse than the others, and as in my first speech at a debating society, "Mr. President" was all that would come out, and brought down the house, so, in my first effort at filling, nothing would stay in, and down came the house again. But the lesson learned as against presumption was Golden.

There was a famous teacher in that earliest school of Dental Science, whose operative theories have been the text and guide of my professional life. He had great celebrity as a dentist, and I resolved to obtain some personal evidence of his skill. By persistence I captured thirteen fillings of the purest gold, and was proud of my achievement. A few months later ten of those fillings vacated their places, and the remaining three had no saving force. It was the old story of good teaching and bad practice, told then, and which, more the pity, will be repeated while the world endures. Adverse experience did not stop, but rather stimulated effort to advance its lines and make a golden progress. After years of preparation I came to a business settlement. My motto was "All for my profession," and my special mission "Salvation to human teeth." To this end I labored, and was never unmindful of the grandeur of my calling.

One day there came to my office a gentleman whose upper front teeth contained approximal fillings of gold. I had never seen anything to equal them in excellence. This work of a master had kept its place for twenty years. I sought out its author, and made a long journey to find and employ him myself. Having reached his place, I was not long in waiting, when an aged man, emaciated, feeble, and wearing green goggles, appeared before me. This was the executor of that wondrous work, done twenty years before. My courage fell; he had been skillful then, could he be so now, was the question that crossed my mind. I was invited to take the chair, when my fears were increased by seeing him *sit down* to his work. Weary and wan he was, but faithful ever. He could do no otherwise than well. It was his inspiration and his habit, and he had not changed. A few hours spent with him brought goodly advance in knowledge. His was the best practical school I had ever attended. By the same acts he saved, taught me to save, and impressed ideas worthy of adoption regarding desert and the rewards for merit.

A youthful alumnus, having gone through his legally prescribed studies, receives the addendum of D. D. S., which signifies only that he has been in the way of learning. But to him whose work has been perfected through much experience and trial, that title belongs with a *post attaindi* extension, D. D. S., H. A., "Doctor of Dental Surgery, Having Attained." The dental father whose labors were blessings to me, deserved these titles well. Those feeble hands have laid their burthen down. The faithful worker has gone to his rest. He sleeps in Greenwood now, and his record is Golden.

Through the singular conduct of a professional brother my next step forward was made. This gentleman had come on a visit to the place of my residence, and I requested him to fill a frail lateral incisor. For some reason he did not take kindly to the operation. In fact he was angry, and manifested that disposition by the roughness of his hand. Up to that time I did not know how much of force a weak tooth could stand. But when he had finished, timidity was gone, and my confidence in the strength of dental tissues was greatly increased. The rough hand and anger had made a revelation, and my perceptions were Golden.

About that time a merchant from New Orleans called to see if I would fill a cavity temporarily, until he could go to his favorite dentist in New York. I did not temporize, but while my work was under way glanced from time to time at a right lateral incisor, decayed almost to exhaustion, and enquired why he did not have it saved. "Oh," was the reply, "that tooth has been a great cross in my life. My dentist says it cannot be filled, and advises leaving it to fall off—after which he proposes to place an artificial crown upon its root." If it were possible, would you like to have it saved? "Indeed I would," he said, "and be ever grateful for so valuable a service." I took the contract, and executed it with entire success. That man came to temporize, but all his teeth were permanently saved, his life-long friendship secured, and my triumph was Golden.

One more incident, and our field of observation shall change. After my coming to New York, a bachelor consulted me in reference to a difficult cavity in one of his upper front teeth, which was always losing its filling. It seemed to have no retaining capacity, although several professional *élites* had done their best for it. I was questioned as to the possibility of making anything stay, and promised to try. The great had failed, and I was ambitious to win. My work had been a fixture for eight years, when that bachelor suddenly appeared before me with the abrupt declaration that my filling had come out. Becoming conscious of an indiscretion, he attempted to lessen its force by adding, "It was not your fault; the filling remained longer than all its predecessors and is amply excused." I did not admit the vacation, but proceeded to try the case while standing at my chair. "You noticed the filling loose in your mouth and removed it thence with your thumb and finger?" Oh, no, he had not *seen* it, and supposed it was swallowed while eating. "On your way to my office cold air struck the cavity and gave you pain?" He had felt no pain, and for fear of trouble had hastened to tell me what had happened. "How did you know the filling was out?" He had recently been married, and desiring to transfer the care of his wife's teeth to my hands, told her the story of his dental experience, dwelt with emphasis upon what I had done, and requested her to look, on doing which she exclaimed, "Why, I see a black hole there now." All the evidence being in, I decided no change had occurred, and that the cavity was still full. The filling was an approximal one and, shaded by a slightly projecting canine, appeared dark upon its surface, but when seen in the light was yellow and Golden.

These simple incidents, these accumulative links in the chain of progress may have failed to interest you, yet out of them and their like has grown the practice I represent to-night. Given a full denture of middle age and average natural strength, with adverse impingements and lateral contacts, with retaining pockets and other accessories to decay; given the crown and approximal cavities usually accompanying these conditions, what shall be done most effectually to save such teeth ? "Fill them with gold," you say, but this does not settle the matter. The problem is too serious to solve in that way alone. Its solution continually goes on, and is seldom completed. I apprehend that failures come mainly from wrong beginnings. We do not study cause sufficiently, and are astonished at effects. Let us begin right now. In the name of common sense, what is the first inducing cause of decay between teeth ? Believe your own senses, and say contact. Why contact ? Because the touching of two teeth, with the gum as a base, makes a triangular pocket which catches and retains foreign matter and sets up a chemical laboratory, wherein are generated acids that destroy the dental tissues. What pockets do for the sides of teeth, crevice and fissure accomplish for their crowns. If, then, the dentures you desire to save are sorely beset, and you know the sources and direction of attack, do not let fossilized ideas or the rut of precedence restrain your action. Rise once for all above the fallacy that enamel is an essential protection against decay. In smoothness alone has it any right to this claim? Its intent, mainly, is to avoid sensitiveness and resist abrasion. If contact is inimical, then change it for a friendly condition, and take the first righteous step toward salvation. Separate freely, and prepare the way for excavation and filling. Make plenty of room. Separation, in its best form, is a combination of the approximal arch and lingual bevel. The one maintains acquired space, the other repels extraneous deposits, and altogether facilitate operations and subsequent care.

Proceed without doubting. There is no occasion to cut down and waste by those terrible slots that reach from turret to base, from crown to foundation. No need to deface the beautiful structure which adorns, while yet it conceals. So excavate, if possible, that when your fillings are in place, no eye will discover that your hand has been there. In your cavities make honest, fast-holding undercuts, and as you are filling with gold, don't deceive yourselves by eschewing retainers. They are absolutely essential to good work. Use adhesive gold always, and be grateful that such a blessing exists. With fine serrations pack in by hand pressure. Condense as you progress, and in nowise neglect at the close to burnish finely and firmly. This plan, explained in brief, involves the æsthetic sense and highest expression of dental art. Saving fillings of gold are extremely rare. They are as one to a thousand, and shine out amid encumbrance and dross like bright particular stars from a darkened sky.

Oh, gold! Oh, virgin gold! What desecrations are committed in thy name. The fillings that fall out and confess are respectable as compared with those that stay in and deceive! Indelicate obtrusion and garish show usurp the places of retiring excellence and modest worth. Jack-screws, and clamps, and mallets of various device, lead on the assault, and nature in the vestibule of her most beautiful temple is subjected to violence. Three generations have given me their faith. Three generations have received the best in my power to bestow, and they ask me now to whom they shall go when my labors are done. I cannot reply. But life, and love, and plenty, and favor abide with me still—for saving is Golden!

